# Ischemic Necrosis of the Gastric Remnant 42 Years After Bariatric Surgery

**DOI:** 10.31486/toj.25.0031

**Published:** 2025

**Authors:** Natalie L. Whitfield, Kyle J. Hoffman

**Affiliations:** ^1^ Rocky Vista University Colorado, Englewood, CO; ^2^ Department of General Surgery, Memorial Hospital of Sweetwater County, Rock Springs, WY

**Keywords:** *Bariatric surgery*, *complications*, *gastric bypass*, *gastric stump*, *necrosis*, *thrombosis*

## Abstract

**Background:**

Ischemic necrosis of the gastric remnant following Roux-en-Y gastric bypass (RYGB) surgery is a rare but life-threatening complication. The etiology remains poorly understood, and delayed presentation can result in poor outcomes. Prompt recognition and intervention are critical, particularly in patients with a remote history of bariatric surgery.

**Case Report:**

A 78-year-old male (body mass index 28.73 kg/m^2^) with a history of RYGB performed 42 years prior presented to the emergency department with nausea, diffuse abdominal pain, and signs of septic shock. Physical examination revealed abdominal distension, peritonitis, tachycardia, and hypotension. Laboratory findings showed elevated lactate and lipase levels. Imaging demonstrated a dilated gastric remnant and free intra-abdominal fluid. Emergent exploratory laparotomy identified a necrotic gastric remnant, necessitating remnant gastrectomy. The patient initially stabilized postoperatively but developed pneumonia on postoperative day 6, followed by a duodenal stump leak. Despite conservative management, his condition deteriorated, and comfort care measures were initiated. He died on postoperative day 13.

**Conclusion:**

This case highlights the importance of maintaining a high index of suspicion for rare complications such as gastric remnant necrosis in patients with a history of RYGB and a low threshold for surgical exploration. Timely diagnosis and surgical intervention are essential for improving outcomes in these critically ill patients.

## INTRODUCTION

Ischemic necrosis of the gastric remnant following Roux-en-Y gastric bypass (RYGB) surgery is an extremely rare but serious complication, with only a limited number of case reports available in the literature.^1-5^ Gastric remnant necrosis occurs when the blood supply to the remnant stomach is compromised, either by decreased blood flow or thrombosis, leading to ischemia and subsequent necrosis. Necrosis of the gastric remnant is a rare occurrence because of the stomach's robust blood supply.

The nonspecific symptoms—tachycardia, hypotension, and diffuse abdominal pain—mimic common postoperative complications such as small bowel obstruction and complicate timely diagnosis. Gastric remnant necrosis is associated with high morbidity and can quickly progress to life-threatening complications if not promptly addressed.

## CASE REPORT

A 78-year-old male (body mass index 28.73 kg/m^2^) was brought by ambulance to the emergency department (ED) with nausea, diffuse abdominal pain, and signs of septic shock. The patient's medical history included RYGB performed 42 years prior to presentation, atrial fibrillation, and hypertension. The atrial fibrillation and hypertension had been previously managed by his primary care physician. The patient had presented to the ED 2 years prior with similar symptoms, was found to have a pseudoaneurysm of the left gastric artery, and received coiling for the pseudoaneurysm.

Physical examination revealed abdominal distension, peritonitis, tachycardia (123 beats per minute), and hypotension (90/65 mm Hg). Abnormal initial laboratory values included lactate of 5.8 mmol/L (reference range, 0.5-2.2 mmol/L), lipase of 1,198 U/L (reference range, 0-160 U/L), and total bilirubin of 1.9 mg/dL (reference range, 0.1-1.2 mg/dL).

**Figure 1.**
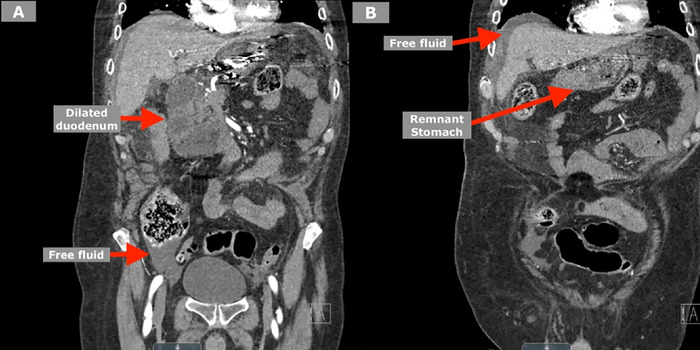
Computed tomography scans in the coronal plane show (A) the dilated duodenum with free fluid beneath the colon and (B) the dilated remnant stomach with free fluid surrounding the liver.

Computed tomography (CT) of the abdomen and pelvis indicated a dilated remnant stomach, severely dilated duodenum, dilated loops of small bowel in the left upper quadrant, and free fluid throughout the abdomen (Figure 1). Given the septic shock and imaging findings, the patient was taken emergently to the operating room for exploratory laparotomy and resection of the gastric remnant.

**Figure 2.**
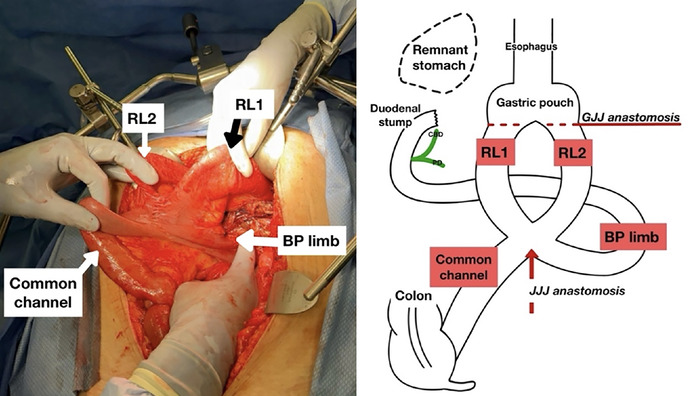
**Photo and illustration of the patient's atypical anatomy.** BP limb, biliopancreatic limb; CBD, common bile duct; GJJ, gastrojejunojejunal; JJJ, jejunojejunal; PD, pancreatic duct; RL1, Roux limb 1; RL2, Roux limb 2.

During the procedure, the patient's vasopressor requirements escalated. Because of his intraoperative instability, a 3M AbThera wound vacuum (Solventum Corporation) was placed, and the patient was transferred to the intensive care unit for further management. He responded well to resuscitation overnight and underwent abdominal closure the following day. A retrograde duodenostomy tube was placed to protect the duodenal stump, and a falciform flap was placed over the stump to reinforce it. A jejunostomy tube was placed for enteral access. During this procedure, the patient's atypical anatomic structure was discovered. He had 2 Roux limbs connecting to the gastric pouch in an antecolic fashion, and the 2 Roux limbs were connected to the biliopancreatic limb with a jejunojejunal anastomosis (Figure 2).

Initially, the patient improved. He was extubated, tolerated feeds, had good urine output, and required only 2 liters of oxygen via nasal cannula. However, on postoperative day (POD) 6, his condition deteriorated, and he was reintubated after being diagnosed with pneumonia. CT scan on POD 9 revealed a duodenal stump leak tracking through the midline fascia, without evidence of free intraperitoneal spillage.

Despite supportive care, including antibiotics and wound management, the patient's condition continued to decline. After discussions with the family, the patient was transitioned to comfort care measures, he was palliatively extubated, and he died on POD 13.

## DISCUSSION

Several theories have been proposed to explain gastric remnant necrosis following RYGB, including small bowel obstruction caused by adhesions and stenosis leading to dilation of the gastric remnant and subsequent ischemia.^1-7^ Other contributing factors include prolonged use of nonsteroidal anti-inflammatory drugs (NSAIDs), smoking, alcohol consumption, and *Helicobacter pylori* infection, all of which can cause mucosal ischemia and ulceration leading to perforation.^3,4,7^ Another hypothesis suggests that surgical manipulation of the gastric vessels—such as ligation of the short gastric arteries or branches of the left gastric artery—may compromise blood flow to the remnant stomach and increase susceptibility to ischemia.^3^

In our patient, small bowel obstruction and stenosis were ruled out, as both the jejunojejunal and gastrojejunojejunal anastomoses were widely patent. Because of the lack of histologic evidence of *H pylori* and because no ulceration was shown on a recent endoscopy, vascular compromise from surgical manipulation, thrombosis, or atherosclerotic disease was the likely etiology.

After RYGB, the remnant stomach is typically supplied only by the right gastric and right gastroepiploic arteries because the left gastric, left gastroepiploic, and short gastric arteries are divided. However, our patient's atypical anatomy and the potential differences in surgical techniques 42 years ago are important considerations. These factors could have altered the expected vascular supply and increased the vulnerability to ischemia.

Given the patient's history of atrial fibrillation and hypertension, we hypothesized that thrombosis or hypoperfusion of the right gastric artery led to necrosis. The patient's left gastric artery pseudoaneurysm, treated 2 years prior by coiling, suggests prior vascular compromise. An earlier ischemic insult in the remnant could have led to chronic inflammation, pseudoaneurysm formation, and eventual necrosis. The pseudoaneurysm formation likely reflected underlying inflammatory changes and chronic ischemia at the staple line of the gastric remnant, which corresponded to the most necrotic area observed during resection.

The limited literature emphasizes the importance of considering gastric remnant necrosis in patients with a history of bariatric surgery who present with signs of shock. Prompt recognition, source control, and timely surgical intervention—including resection of necrotic tissue—are critical for improving outcomes.

## CONCLUSION

Although complications from RYGB are well documented, ischemic necrosis of the gastric remnant—particularly in the absence of small bowel obstruction or ulceration attributable to *H pylori* infection or excessive NSAID use—is rare. This case highlights the need to consider vascular compromise in patients with prior bariatric surgery who present with abdominal sepsis or unexplained shock. Atypical anatomy, previous vascular events, and altered blood supply may increase the remnant stomach's susceptibility to ischemia. Given the rarity of this complication, further investigation and case documentation are necessary to better understand the underlying mechanisms and risk factors for gastric remnant necrosis after RYGB.

## Notes

*This article meets the Accreditation Council for Graduate Medical Education and the American Board of Medical Specialties Maintenance of Certification competencies for Patient Care and Medical Knowledge.*
